# Cocaine Intoxication

**Published:** 1886-12

**Authors:** 


LESTER CURTIS.

---

COCAINE INTOXICATION.

Dr. Commanus, Berlin,* describes a case of cocaine intoxication. The patient had contracted the morphine habit in seeking relief from hemorrhoids, and used cocaine as a substitute for morphine. He began with 0.05 grm. of muriate of cocaine three or four times daily. The remedy at first gave him real comfort without producing after effects worth mentioning. According to his account he enjoyed an alleviation of his hemorrhoidal difficulty and had a stool every day, which was not the case when using the morphine.

He finally reached doses of 0.5 to 0.8 grm. daily, when he began to suffer with "a poor appetite, ringing in the ears, occasional shortness of breath, and hallucinations in respect to the senses of sight and hearing." These symptoms he learned how to remove by small doses of morphine.

During an attack of herpes zoster he used doses of from a gramme to a gramme and a half daily for two or three days. Then followed "trembling of the limbs, relaxation of the muscles of the body, peculiar and rapidly extending

* Berliner Klinische Wochenschrift, September 20th, 1886.

changes of the finger and toe nails, loss of appetite and sleep, very great agitation, strong hallucinations in the departments of the nerves of sight, smell and hearing; intensely injected conjunctivæ; a staring look. The patient fired several revolver shots at the objects of his hallucinations. He attacked his servant in order to force out of his mouth a lantern which was concealed there." These symptoms are seen to resemble those of delirium tremens. They were relieved in two or three days by small doses of morphine.

Dr. Mærkel,* also gives some warnings as to the danger of using cocaine.

Among these is the " significant sleeplessness lasting up to six hours after the cocaine injection."

He doubts, however, the danger of forming a cocaine habit, like the morphine habit, because he does not believe that cocaine is a pure nervine as morphine is. It produces its effect first through the nutrient grandular system, which it stimulates' causing an excess of nutrition to flow to the nerve centres. His reasons for this opinion are that after the use of cocaine, a certain amount of salivation is always produced. There always follows a swelling of the lymphatic glands in spite of the most careful antiseptic precautions; even the mammary gland is sometimes affected in the male. This result is more marked on the side which has received the injection.

The appetite is always increased as after a drain upon the nutrition.

The drug produces not so much a stimulation as a feeling of well-being and elasticity, a disposition to play, as in growing young animals. After the effect has passed off there is no

* Berliner Klinische Wochenschrift, March 8, 1886.

" katzenjammer," as after a debauch, only a return to the usual condition. At the most there is only a feeling of pleasant weakness.

It is in this disturbance of nutrition that he thinks the danger in its use lies. As a partial proof of this view, he states that the cocaine users of Central America always die of phthisis. LESTER CURTIS.

